# Supplementary material for: Migration Pattern, Habitat Use, and Conservation Status of the Eastern Common Crane (Grus grus lilfordi) from Eastern Mongolia
Source: Animals (Basel). 2023 Jul 12;13(14):2287. doi: 10.3390/ani13142287 (PMC10375961; doi:10.3390/ani13142287)
Supplement: Supplementary file 1 [file animals-13-02287-s001.zip › animals-2430220-supplementary.pdf]

Migration Pattern, Habitat Use, and Conservation Status of the Eastern Common Crane (*Grus grus lilfordi*) from Eastern Mongolia

Supplementary materials

Table S1. Basic information of the 11 Eastern Common Cranes *G. g. lilfordi* captured in Mongolia and fitted with a satellite transmitter.

Bird ID	Capture date	Capture site	Age	Sex	Cranes body weight (g)	Total number of locations	Duration of generating data*
CC116	25 Jul, 2017	Chukh Lake	Juv	-	3295	22,152	25 Jul, 2017-6 Apr, 2021
CC185	24 Jul, 2017	Galuut Lake	Juv	-	3735	788	31 Jul, 2017-20 Jul, 2018
CC119	28 Jul, 2017	Chukh Lake	Juv	-	3825	4,033	28 Jul, 2017-16 Oct, 2020
CC210	25 May, 2018	Turgenii tsagaan Lake	SubAd	Female	4215	5,498	25 May, 2018-12 Apr, 2019
CC209	28 May, 2018	Turgenii tsagaan Lake	Ad	Male	4985	6,576	30, May, 2018-9 Apr, 2019
CC213	2 Jun, 2018	Turgenii tsagaan Lake	Ad	Male	5125	7,499	2 Jun, 2018-16 Apr, 2019
CC201	22 Jul, 2019	Chukh Lake	Juv	-	3335	4,145	22 Jul, 2019-14 Mar, 2020
CC061	22 Jul, 2019	Chukh Lake	Juv	-	3410	8,765	22 Jul, 2019-11 Jul, 2020
CC277	1 Aug, 2019	Turgenii tsagaan Lake	Juv	-	2600	19,791	1 Aug, 2019-26 Nov, 2021
CC279	2 Aug, 2019	Kholboo Lake	Juv	-	4050	17,235	2 Aug, 2019-19 Oct, 2021
CC281	2 Aug, 2019	Kholboo Lake	Juv	-	4050	12,742	2 Aug, 2019-26 Apr, 2021

* Cut-off date：The collected data used in this study is cut from captured date to until 26 Nov 2021.

Table S2. Migration parameters of the 11 Eastern Common Cranes *G. g. lilfordi* on 36 spring/autumn migration trips.

Bird ID	Season and year	Departure date	Arrival date	Migration duration (day)	Stopover duration (day)	Travel duration (day)	Migration distance (km)	Migration speed (km/day)	Travel speed (km/day)	Number of the stopover
CC116	Autumn, 2017	19 Sep, 2017	25 Nov, 2017	67	65	2	1933	29	967	2
	Spring, 2018	25 Feb, 2018	5 Apr, 2018	39	37	2	1767	45	884	2
	Autumn, 2018	3 Sep, 2018	29 Nov, 2018	87	85	3	2637	30	879	2
	Spring, 2019	28 Feb, 2019	22 Apr, 2019	53	41	12	2934	55	245	1
	Autumn, 2019	17 Sep, 2019	20 Nov, 2019	64	61	3	2207	34	736	2
	Spring, 2020	5 Mar, 2020	8 Apr, 2020	34	30	4	2344	69	586	1
	Autumn, 2020	18 Sep, 2020	30 Nov, 2020	72	69	3	2097	29	699	2
	Spring, 2021	27 Feb, 2021	6 Apr, 2021	38	35	3	1869	49	623	2
CC185	Autumn, 2017	17 Sep, 2017	23 Oct, 2017	36	13	-	1199	33	-	1
	Spring, 2018	24 Apr, 2018	30 Jul, 2018	40	38	2	1919	48	960	2
CC119	Autumn, 2017	23 Sep, 2017	3 Dec, 2018	71	69	2	1744	25	872	2
	Spring, 2018	5 Mar, 2018	6 May, 2018	62	60	2	1971	32	986	2
	Autumn, 2018	12 Sep, 2018	29 Oct, 2018	47	45	2	1223	26	612	1
	Spring, 2019	9 Apr, 2019	12 Apr, 2019	3	0	3	1138	379	379	0

	Autumn, 2019	9 Sep, 2019	2 Nov, 2019	54	51	3	1206	22	402	1
CC210	Autumn, 2018	3 Sep, 2018	27 Oct, 2018	54	52	2	1420	26	710	1
	Spring, 2019	10 Apr, 2019	12 Apr, 2019*	-	-	-	987	-	-	0
CC209	Autumn, 2018	16 Sep, 2018	28 Nov, 2018	73	71	2	2079	29	1040	2
	Spring, 2019	3 Mar, 2019	9 Apr, 2019*	37	33	4	1739	47	435	1
CC213	Autumn, 2018	30 Sep, 2018	24 Oct, 2018	24	22	2	1267	53	634	1
	Spring, 2019	1 Apr, 2019	16 Apr, 2019*	15	13	2	957	64	479	1
CC201	Autumn, 2019	23 Sep, 2019	30 Dec, 2019	98	95	3	1770	18	590	2
CC061	Autumn, 2019	23 Sep, 2019	30 Dec, 2019	98	95	3	1776	18	592	2
	Spring, 2020	23 Feb, 2020	3 May, 2020	70	68	2	1313	19	657	2
	Autumn, 2019	11 Oct, 2019	28 Dec, 2019	78	74	4	1786	23	447	1
CC279	Spring, 2020	21 May, 2020	30 May, 2020	9	8	1	345	38	345	1
	Autumn, 2020	22 Nov, 2020	26 Nov, 2020	4	3	1	504	126	504	1
	Spring, 2021	6 May, 2021	10 May, 2021	4	3	1	489	122	489	1
	Autumn, 2019	11 Oct, 2019	13 Oct, 2019	2	0	2	1242	621	621	0
CC281	Spring, 2020	2 May, 2020	5 May, 2020	3	0	3	1437	479	479	0
	Autumn, 2020	17 Sep, 2020	18 Sep, 2020	1	0	1	1234	1234	1234	0

CC277	Autumn, 2019	17 Sep, 2019	14 Oct, 2019	27	25	2	1337	50	669	1
	Spring, 2020	2 May, 2020	8 May, 2020	6	0	6	1895	316	316	0
	Autumn, 2020	25 Aug, 2020	30 Aug, 2020	5	3	2	1570	314	785	1
	Spring, 2021	10 Apr, 2021	25 Apr, 2021	15	13	2	1472	98	736	2
	Autumn, 2021	8 Sep, 2021	5 Oct, 2021	27	25	2	1400	52	700	2

* Tracking data interrupted.

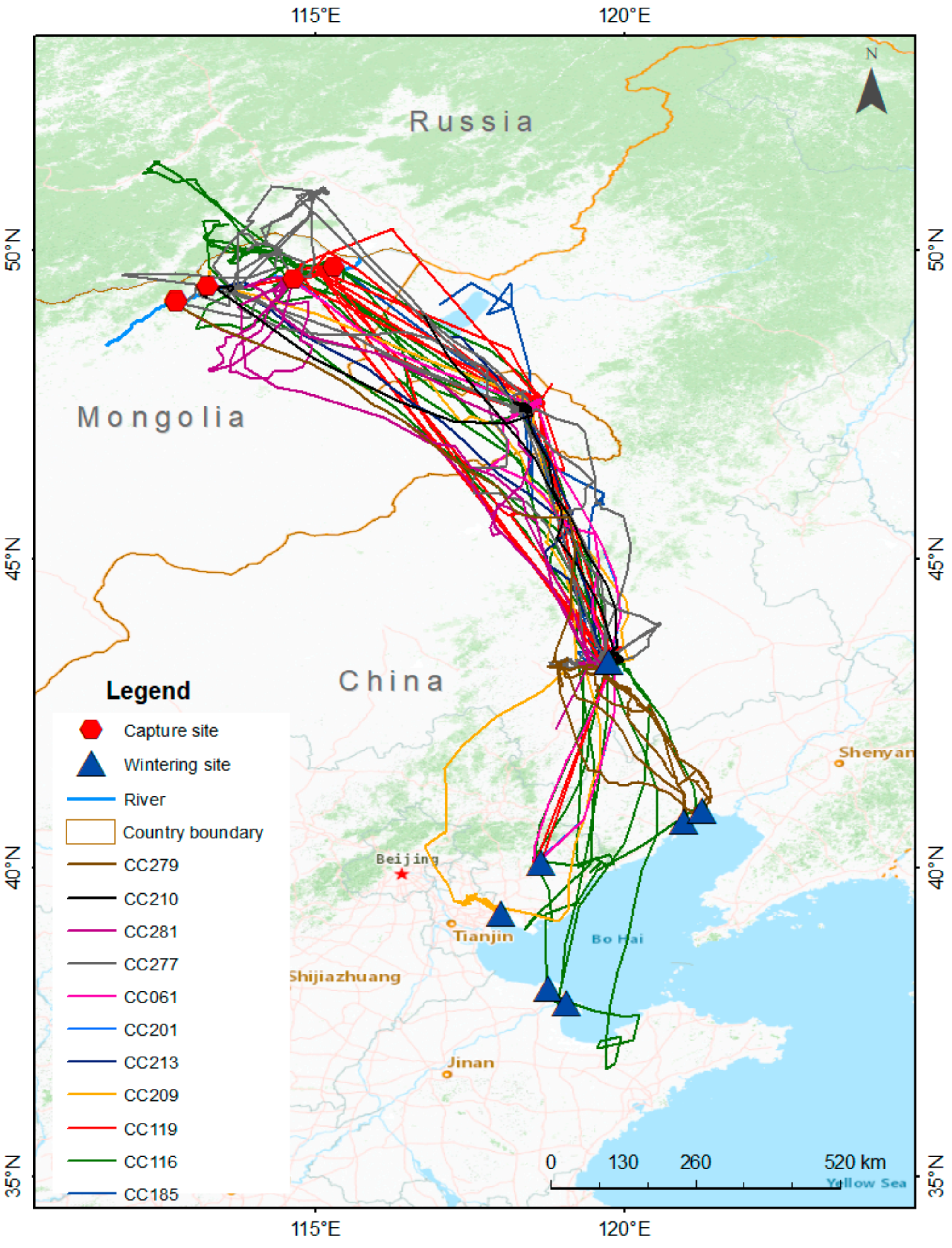

Figure S1. Migration routes and critical sites of the 11 Eastern Common Cranes *G. g. lilfordi*.
